# Supplementary material for: Health of children who experienced Australian immigration detention
Source: PLoS One. 2023 Mar 9;18(3):e0282798. doi: 10.1371/journal.pone.0282798 (PMC9997934; doi:10.1371/journal.pone.0282798)
Supplement: Attachment — Submitted filename: Review_PONE-D-22-29610.pdf [file pone.0282798.s001.pdf]

PONE-D-22-29610
Manuscript Review
November 13, 2022

Summary of the revision and overall impression

The study tackles an important public health problem, the impact of held detention on the health and wellbeing of children and adolescents. This cross-sectional study used medical records from the Royal Children´s Hospital Immigrant Health Service, with the aim of describing the physical and mental health status of children who experienced held detention in Australia. One of the main strengths of the study is the data sources used (e.g., clinical records). For me, the strength of these type of data sources is that it can be seen as a more objective measure of health outcomes, when compared to self-reporting, which is a methodology often used in studies examining health outcomes of migrant populations. I would only suggest the authors to make a stronger case for the gap in the literature regarding the impact of detention centers on children's health, by highlighting the methodology used. There are studies, mainly carried out in the United States, about the impact of detention centers on children´s health. However, as mentioned above, most use self-reported measures to evaluate the impact.

Major and minor issues

a) Major issues

- No major issues.

b) Minor issues:

1. General comments:

- I recommend language editing of the manuscript. The ideas presented in some paragraphs can be made clearer for the readers with minor language editing of the manuscript.

2. Introduction:

- It would be helpful for readers who are not familiarized with the Australian immigration system to have a better understanding in the introduction of the referral process to the Royal Children´s Hospital Immigrant Health Service. Why do children get referred to these health services? Are all children in detention receiving health services in this hospital or only those children who are referred by the immigration system? If this is the case, why some children get referred and why some children do not get referred to these health services? What are the criteria being used?
- It would also be helpful to know more about the differences between the Immigration Detention Centers on Australian mainland and the offshore Regional Processing Centers

in Nauru and Manus Island, Papua New Guinea. What are the characteristics and processes that make these detention centers different from each other?

3. Methods:

- It would be useful for readers if authors described why they decided to analyze the health outcomes separately for the Immigration Detention Centers on Australian mainland and the offshore Regional Processing Centers in Nauru and Manus Island. This could be tied to the introduction suggestion of describing how are these immigration detention centers different.

4. Results:

- Include a statement like “results not shown in tables” for those results who are not included in descriptive tables.
- I would suggest to only include the percentages in the parentheses, not both the percentages and the number of participants. Maybe just leave the n´s when the total n is different from what is previously stated in that paragraph. For example, delete here: “Almost half (43%, n=120/277) the cohort had sleep difficulties, 27% (n=76/277) reported nightmares and 10% (n=28/277) of children had engaged in self-harm”. But keep it here: “Attachment issues with severe functional impact were diagnosed in 53% (n = 16/31) of infants born in detention….”.

5. Discussion

- It is not clear in the manuscript at which point during the detention process children receive medical care in the Royal Children´s Hospital Immigrant Health Service. A section in the discussion about this would be helpful to readers to understand if the health outcomes are associated to their stay in the immigration detention centers or if they are a result of the migration process. For instance, if children are being evaluated in their first week after arrival to the detention centers, are the nutritional deficiencies associated to reduced access to nutritious food in the detention centers or are they associated to food insecurity in their countries of origin and during the migration process (e.g., before their arrival to the detention centers). I believe that integrating this into the discussion, and/or in the results section, could strengthen the argument the authors are making.
- Discuss the differences found when comparing health outcomes between the Immigration Detention Centers on Australian mainland and the offshore Regional Processing Centers in Nauru and Manus Island, Papua New Guinea. Why are some health outcomes more prevalent in Nauru vs. the Australian mainland detention centers, and vice versa? Are the conditions in these centers different? Could these conditions be impacting differently the health of children and families being detained?
- Discuss how generalizable are the study findings, based on the methodology implemented. Are these results generalizable only to participants in the study? Could

the results be generalizable to other children held in detention in Australia or other parts of the world and why or why not?

6. Figures

- For Figure 1, I would suggest including axis captions for both the x and y axis, as well as a descriptive title.
